# AGC2 (Citrin) Deficiency—From Recognition of the Disease till Construction of Therapeutic Procedures

**DOI:** 10.3390/biom10081100

**Published:** 2020-07-24

**Authors:** Takeyori Saheki, Mitsuaki Moriyama, Aki Funahashi, Eishi Kuroda

**Affiliations:** 1 Department of Hygiene and Health Promotion Medicine, Kagoshima University Graduate School of Medical and Dental Sciences, 8-35-1 Sakuragaoka, Kagoshima 890-8544, Japan; aki.2784@gmail.com (A.F.); kurodaeishi1029@yahoo.co.jp (E.K.); 2 Laboratory of Integrative Physiology in Veterinary Sciences, Osaka Prefecture University, 1-58 Rinku-oraikita, Izumisano, Osaka 598-8531, Japan; moriyama@vet.osakafu-u.ac.jp

**Keywords:** adult-onset type II citrullinemia (CTLN2), aspartate/glutamate carrier (AGC), animal model, argininosuccinate synthetase (ASS), aversion to carbohydrates, citrin, food taste, neonatal intrahepatic cholestasis caused by citrin deficiency (NICCD)

## Abstract

Can you imagine a disease in which intake of an excess amount of sugars or carbohydrates causes hyperammonemia? It is hard to imagine the intake causing hyperammonemia. AGC2 or citrin deficiency shows their symptoms following sugar/carbohydrates intake excess and this disease is now known as a pan-ethnic disease. AGC2 (aspartate glutamate carrier 2) or citrin is a mitochondrial transporter which transports aspartate (Asp) from mitochondria to cytosol in exchange with glutamate (Glu) and H^+^. Asp is originally supplied from mitochondria to cytosol where it is necessary for synthesis of proteins, nucleotides, and urea. In cytosol, Asp can be synthesized from oxaloacetate and Glu by cytosolic Asp aminotransferase, but oxaloacetate formation is limited by the amount of NAD^+^. This means an increase in NADH causes suppression of Asp formation in the cytosol. Metabolism of carbohydrates and other substances which produce cytosolic NADH such as alcohol and glycerol suppress oxaloacetate formation. It is forced under citrin deficiency since citrin is a member of malate/Asp shuttle. In this review, we will describe history of identification of the *SLC25A13* gene as the causative gene for adult-onset type II citrullinemia (CTLN2), a type of citrin deficiency, pathophysiology of citrin deficiency together with animal models and possible treatments for citrin deficiency newly developing.

## 1. Introduction

AGC2 or citrin deficiency is the most prevalent autosomal recessive inherited metabolic disease in East and East-south Asia and it is now known as a pan-ethnic disease [1,2,3,4,5,6,7,8,9], and is one of the many diseases caused by mutations in the genes encoding members of the SLC25 protein family [10,11]. Its heterozygote frequency in East Asia is up to 1 in 40 [12,13]. There are mainly 2 disease types, CTLN2 (adult-onset type II citrullinemia) and NICCD (neonatal intrahepatic cholestasis caused by citrin deficiency) [14,15,16,17]. Citrin deficiency also causes FTTDCD (failure to thrive with dyslipidemia caused by citrin deficiency) [18], pancreatitis [19], NASH [20,21], and hepatoma [22,23]. In this review article, we will describe AGC2 or citrin deficiency starting from the recognition of the disease, discovery of the disease gene for CTLN2 and to pathophysiology of the disease based on the function of AGC2, and therapeutic procedures derived from analysis of food preference.

## 2. Materials and Methods

Materials and Methods are described in each publication. Human patients and mice are analyzed. Informed consents of all subjects are obtained. Animal experiments were done after getting approval of committee of animal experiment at Kagoshima University, Tokushima Bunri University, and Kumamoto University.

## 3. Study Started from Enzymology of Argininosuccinate Synthetase (ASS)

Saheki et al. started purification of ASS from rat and human liver in 1973 [24] on the assumption that since ASS activity is the lowest among the urea cycle enzymes, ASS may be tightly regulated by some factors. We purified the enzyme to a homogeneity and crystalized, but they could not find any significant regulatory mechanism in the ASS protein. During the research, we have been asked by many clinicians or clinical researchers whether the hepatic ASS activity of their citrullinemic patients is low or not, since the enzyme catalyzed the synthesis of argininosuccinate from citrulline and Asp in the expense of ATP breakdown. Since ASS deficiency is assumed to cause citrullinemia, Saheki et al. examined Km values for citrulline, Asp, and ATP, and quantified the ASS protein amount by using anti-ASS antiserum together with assaying ASS activity. Luckily enough, we noticed there are two types of enzyme abnormality in the patients’ ASS in the liver obtained by biopsy or autopsy almost from the beginning [25]. As you see in Table 1, one group is mainly from neonates, the low ASS activities were from lowered affinities to the substrates, citrulline, Asp, or/and ATP, or enlarged Km values for the substrates. Using autopsied samples, we also examined kidney and brain ASSs, which showed the same abnormalities in the enzyme kinetics. The same abnormalities were found in the cultured skin fibroblast cells [26]. The enzyme amounts are normal (type I) or very minute amounts (type III) are found. These results suggest that the abnormality is in the gene of *ASS1*. Kobayashi et al. [27] found real mutations in the *ASS1* genes. So, this is classical or neonatal citrullinemia (CTLN1). The other group, named as CTLN2 later, consisted from mainly adult patients, showed reduced ASS activities which are explainable with the reduced enzyme amount with normal kinetic properties (type II). ASS activities in the brain, kidney, or cultured fibroblast cells are not different from the control. So that the former should be qualitative abnormality caused by mutations in the *ASS1* gene and the latter is quantitative abnormality. Increased urinary excretion of argininosiccinate [28], the product of ASS reaction, in CTLN2 patients probably suggests normal operation of renal ASS reaction. The number of patients were much larger in the latter group. The symptoms of the latter group are consciousness disturbance and abnormal behavior with hyperammonemia, mild citrullinemia, and mild liver damage. From these enzymological abnormalities and clinical symptoms, we named it adult-onset type II citrullinemia, or CTLN2.

Before we have genetic information of the disease, we have several important information; for example, the patients showed high serum PSTI (pancreatic secretory trypsin inhibitor) [29], which is secreted not from the pancreas, but from the liver [30]. This is considered an early marker of disease onset [31].

In our group, Dr. Keiko Kobayashi et al. [32,33,34] performed molecular genetic analysis on these patients after earlier biochemical analysis on the mRNA and protein and found various mutations in the *ASS1* gene in the former group patients, amino acid substitutions in ASS abnormality in its kinetic parameters, splicing site mutations in ASS abnormality with a very minute enzyme amount. They did similar analyses on the samples from patients with type II citrullinemia [35]. Together with our first analysis showing that no abnormalities in the amount and translatable activity of the mRNA [36,37], we finally concluded that there are no abnormalities in the *ASS1* gene in the latter group. Table 1 shows two distinct types of citrullinemia.

## 4. Pathogenesis of Adult-Onset Type II Citrullinemia

What is the primary defect in CTLN2? The decreased level of ASS protein in the liver of CTLN2 patients is in average 9% of the control level (from almost 0% up to 70%). So far, we have not been able to find out the mechanism of the decrease. We thought that the mechanism is not known but should be caused by genetic abnormality because we have found many patients derived from consanguineous marriage, suggesting that it is an autosomal recessive trait. So, we started genomic analysis using homozygosity mapping with 18 DNA samples from CTLN2 patients derived from consanguineous marriage and DNA samples from control subjects. This homozygosity mapping analysis revealed the disease gene located at chromosome 7q21.3 [38]. The gene mutated in CTLN2 patients were searched for in collaboration with Drs. Steve Scherer and Lap-Che Tui, Toronto, Canada. Finally, Dr. Kobayashi found mutations in *SLC25A13*, which encoded mitochondrial transporter [38], which was published in 1999. The results found showed that among 18 samples from consanguineous marriage contained three heterozygotes, suggesting high prevalence of the disease [38].

A very similar gene, *SLC25A12* [39], encoding aralar reported by del Arco, Spain, was published in 1998. Prof. Ferdinando Palmieri, Italy, proposed collaboration among three groups to elucidate the functions of citrin and aralar. The collaboration was successful to find that the two genes encode mitochondrial membrane aspartate glutamate carrier (AGC) [40]. Both aralar (AGC1) and citrin (AGC2) transports proton and Glu from intermembrane space of mitochondria into mitochondrial matrix and Asp from mitochondria matrix to mitochondrial intermembrane space [40], and both activated by calcium [38,41].

AGC1 (aralar) is expressed mainly in the brain, skeletal muscle, heart, and kidney, while AGC2 (citrin) is located mainly in the liver, kidney and heart. So that AGC1 is the brain/skeletal muscle-type and citrin is the liver-type. More precise distributions of aralar and citrin have been reported by Begum et al. [42] and Amoedo et al. [43].

Citrin deficiency causes CTLN2 (OMIM ID #603471) and NICCD (OMIM ID #605814) [1,14], while aralar deficiency is a rare disease causing global cerebral hypomyelination, developmental arrest, hypotonia, and epilepsy (OMIM ID #612949) [44,45]. Lists of mutations in citrin deficiency and AGC1 deficiency can be found at http://www.hgmd.cf.ac.uk/ac/gene.php?gene=SLC25A13 and 12.

## 5. Discovery of NICCD (Neonatal Intrahepatic Cholestasis Caused by Citrin Deficiency) and Citrin Deficiency Disease Types

Since we have discovered the gene for CTLN2, we were very busy creating a diagnostic system for each mutation found in CTLN2 and found many CTLN2 patients not only in Japan, but also in China [3,4,5]. During this time, three pediatric doctors were interested in neonatal hepatitis or cholestasis during neonates. Prof. Tazawa [15] visited Kagoshima and assayed liver ASS activity of his neonatal patients but without any positive results, but later, we diagnosed with DNA diagnosis that his neonatal transient citrullinemia patients were really suffering from citrin deficiency. At the almost same time, we also diagnosed with DNA diagnosis that the transient citrullinemia patients of Dr. Ohura also had mutations in *SLC25A13* [16]. We also diagnosed the patient of Dr. Tomomasa [17] as a citrin deficiency patient: the patient showed NICCD symptoms at his neonatal period and suffered from CTLN2 later at 16 years old. Thereafter, many NICCD patients have been found in Japan, China, Korea, and other countries [1,6,46,47,48].

Laboratory findings of the patients were positive signs of several neonatal mass screening tests such as tyrosine, phenylalanine, methionine, and threonine. Some showed galactosemia [49,50] and cholestasis and bleeding diathesis [2]. Extremely high serum levels of α-fetoprotein are impressive [1,51]. However, there was almost no increase in blood ammonia.

**Figure 1:**
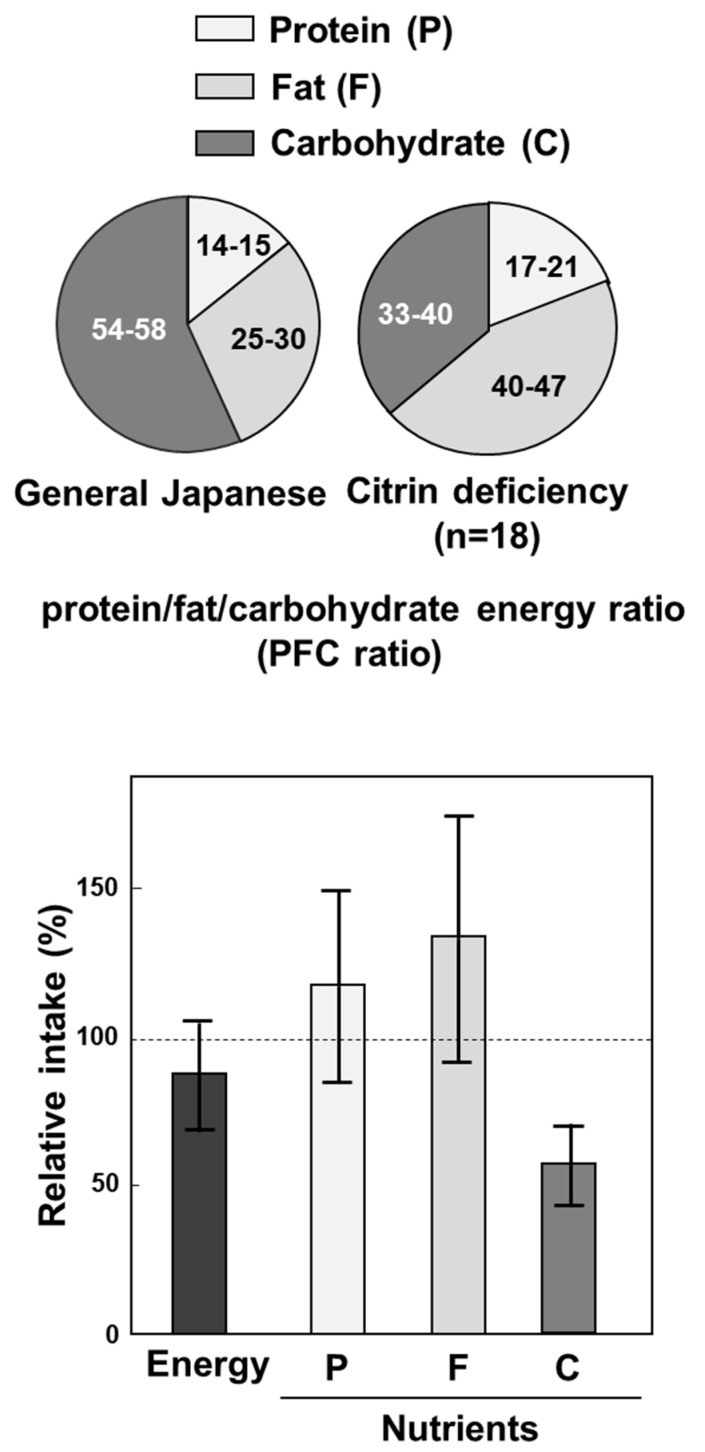
Peculiar food taste of citrin deficiency subjects. Upper panel shows protein, fat, and carbohydrate (PFC) energy ratio, and lower panel shows relative intake of nutrients of general Japanese and citrin deficiency subjects. From [53].

**Figure 2:**
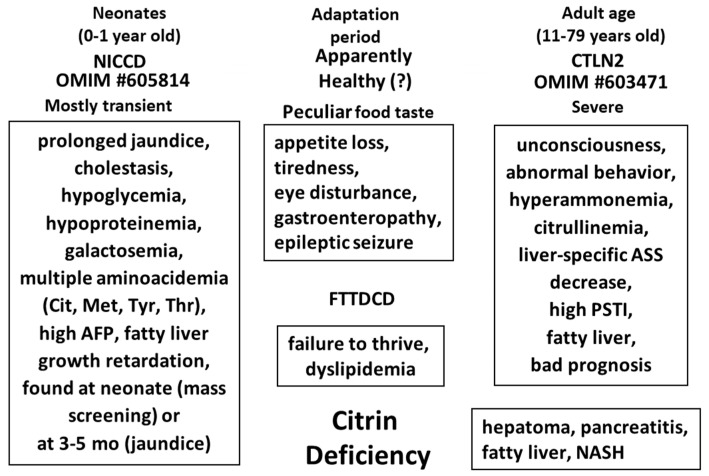
Relation between various disease types during lifetime in citrin deficiency. Modified from [55].

About 50% of NICCD patients were found at neonatal screening and about 40% were at about several months with persistent jaundice [1]. Some were found later with failure to thrive and dyslipidemia (FTTDCC) [18]. Among them, a few, one in 100,000 to 200,000 as the incidence developed to CTLN2 at adult age (10 to 80 years old) with symptoms including hyperammonemia, consciousness disturbance, and abnormal behavior. During the period between NICCD and CTLN2, most of the patients showed almost no severe symptoms except tiredness and almost normal laboratory examinations [52]. However, most of citrin deficiency subjects showed peculiar food taste from about 1 year old. Both our nutritional assessment [53] on symptomless citrin deficiency subjects from 1 year old and 36 years old, and Nakamura et al. on CTLN2 patients [54] revealed reduced intake of carbohydrates, which is very important as a pathogenesis of citrin deficiency and for the treatment of citrin deficiency, as stated later. The peculiar food taste of citrin deficiency subjects is shown in Figure 1. The various disease types of citrin deficiency during lifetime are shown in Figure 2.

## 6. The Metabolic Functions of Citrin and Disease Model

**Figure 3:**
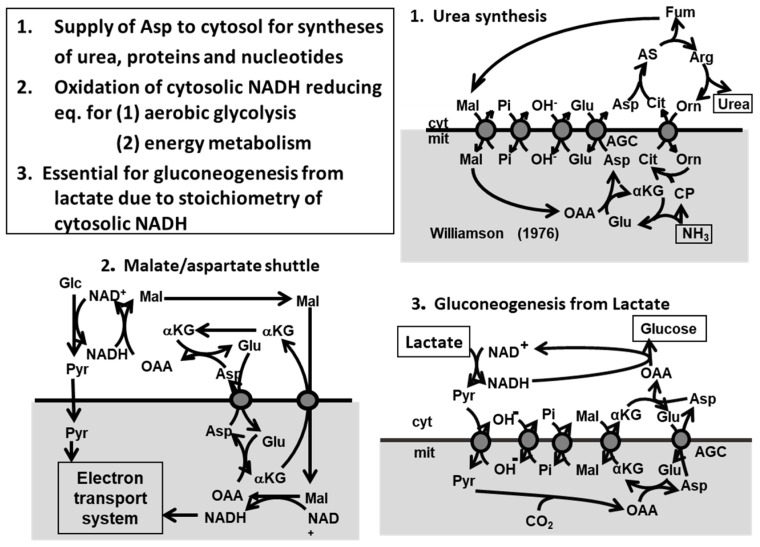
Metabolic functions of citrin. From [57].

The metabolic functions of citrin [56] were shown in Figure 3; (1) as a member of malate/Asp shuttle, it is important for transport of reducing equivalent of NADH into mitochondria which is related to production of ATP in the mitochondria; (2) it plays a role in transport of Asp from mitochondria to cytosol for synthesis of proteins, nucleotides, and urea; (3) it is indispensable for metabolism of lactate to glucose (gluconeogenesis) in order to avoid excess formation of NADH in the cytosol because of stoichiometry of NADH. Mutations of *SLC25A13* gene disturb the metabolic functions listed above.

We have created, at first, citrin gene knockout mice [58] as a model animal, which showed metabolic defects due to citrin-KO in vitro, but with almost no symptoms in vivo. The reason why the mice showed almost no symptoms, we postulated that the rodent liver contains another NADH shuttle, glycerophosphate shuttle, which compensates the defect of malate/Asp shuttle. So, we created double gene knockout mice, which lack not only *SLC25A13*, but also *mitochondrial glycerol 3-phosphate dehydrogenase* (*Gpd2*; mGPD), a member of glycerophosphate shuttle. The resultant citrin/mGPD double-KO mice recapitulated human citrin deficiency [59], showing hyperammonemia, citrullinemia, hypoglycemia, and growth retardation.

## 7. Pathophysiology of the Double-KO Mice

**Figure 4:**
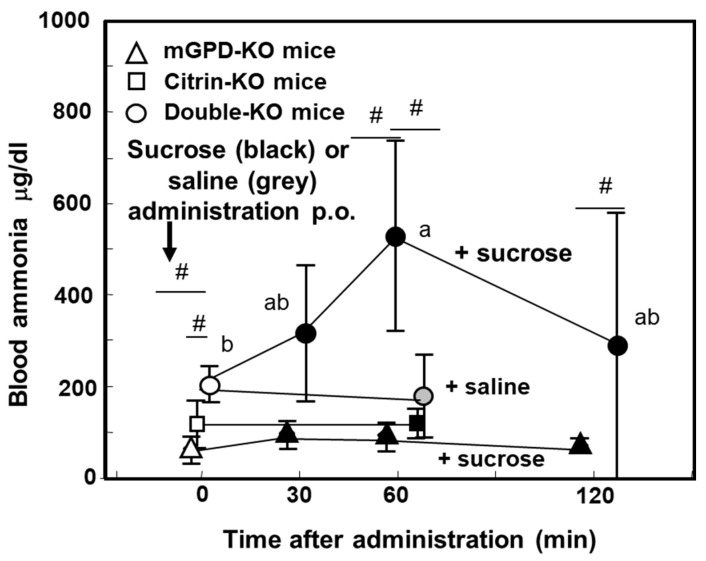
Effect of sucrose or NaCl administration on blood ammonia on fed mGPD-KO, citrin-KO, and double-KO mice. # p > 0.05 from value with Saline. Values with same character (a, ab) indicate no statistical difference. From [57].

**Figure 5:**
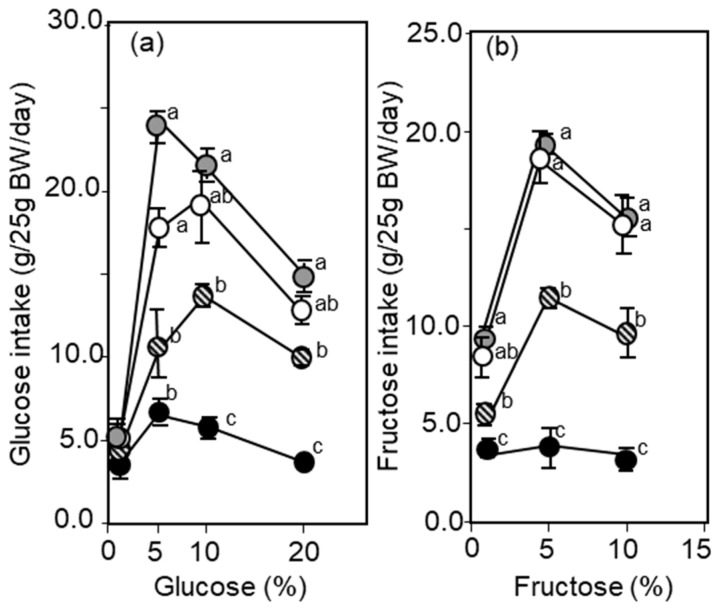
Voluntary intake of various concentrations of glucose (**a**) or fructose (**b**) and water (two bottles, water and a test solution) given to four kinds of mice, wild type (white circle), mGPD-KO (circle with stripes), citrin-KO (grey circle), and double-KO (black circle) were monitored. Concentrations are given in the figure. Intakes were compared statistically and differences were expressed with a, b, c. The same character indicates no difference. It is noteworthy that the double-KO mice took only a small amount of glucose and fructose solutions, as compared with other mice. From [59].

The double-KO mice showed hyperammonemia under fed conditions, but not under starved conditions [59] (Figure 4). Most characteristics is that the double-KO mice showed enhanced severe hyperammonemia when the mice were given sucrose solution forced per os (Figure 4). Furthermore, they did not like to take sucrose solution, as compared with other genotype mice including citrin-KO and mGPD-KO mice. We have shown that not only sucrose, but also, the components, both glucose and fructose [60], had the same effect, as shown in Figure 5. We propose that carbohydrate intake increases cytosolic NADH, which is the reason why citrin deficiency subjects dislike to take carbohydrates. Metabolomic analysis of the double-KO mice liver revealed six major metabolic abnormalities listed as follows [61], (1) a vast increase in glycerol 3-phosphate and ratio of G3P/dihydroxyacetonephosphate, (2) increased concentration of lysine caused by a decreased availability of α-ketoglutarate, resulting in inhibition of breakdown of lysine, (3) increase of citrulline, (4) decreased concentration of Glu, (5) ATP, and (6) citrate. A marked increase in glycerol 3-phosphate (G3P), or G3P/dihyroxyacetone phosphate ratio indicates an increase in the NADH/NAD^+^ ratio in the cytosolic compartment of the liver. This was caused by destruction of two NADH shuttles leading to accumulation of cytosolic NADH, which caused inhibition of glycolytic enzyme, glyceraldehyde 3-phosphate dehydrogenase. The increased NADH also suppressed oxaloacetate formation from malate and malate dehydrogenase, further formation of Asp by cytosolic Asp aminotransferase, together with defect of Asp supply from mitochondria [40], causing inhibition of ASS reaction, resulting in accumulation of citrulline and ammonia, and inhibition of urea synthesis. We postulate that decreased Glu comes from oxidation of NADH in the mitochondria by inactive malate/Asp shuttle and decreased TCA cycle activity, evidenced by low concentration of TCA cycle intermediates [59]. Decreased citrate comes from inhibition of glycolysis and cytosolic increase in NADH/NAD^+^. ATP will decrease by inhibition of glycolysis and inactive malate/Asp shuttle.

Voluntary intake test of sucrose, ethanol, and glycerol [60], of which intakes or administration caused onset or worsening of disease [1,62], revealed that the double-KO mice severely avoided the intakes, citrin-KO and mGPD-KO mice moderately avoided taking the higher concentration solutions of ethanol and glycerol. We found that high correlation between avoidance of intake of the solutions scored from the amounts of intake and comparison between the mice, and simultaneous hepatic increase in G3P and decrease in ATP [60]. We suggest that the two parameters, simultaneous increase in the NADH/NAD^+^ ratio represented by the G3P concentration and decrease in ATP in the liver, send a signal to the brain to induce to suppress the intake of the solution or the foods which increase in hepatic cytosolic NADH and decrease in ATP. This consideration is important from a viewpoint of therapy of citrin deficiency. Namely, correction of either increased G3P or decreased ATP may stop the symptoms of citrin deficiency.

## 8. Treatment of Citrin Deficiency Based on the Pathophysiology of the Disease

Principle concept of citrin deficiency treatment should be based on the peculiar food intake tendency except that liver transplantation is the most effective in correcting all the metabolic disturbances [63,64], liver transplantation at early stage of the disease, almost all symptoms disappeared, suggesting that it is liver disease. Liver transplantation also normalizes the peculiar food taste (Kobayashi, unpublished data). Liver transplantation itself has some drawback, such as the cost and shortage in liver donors and not all the liver transplantation could help the lives of citrin deficiency patients probably due to immunological complications or other causes. Therefore, nutritional and medicinal therapeutic procedures are important. Basics of the therapy is nutrition. High carbohydrates diet causes anorexia in NICCD patients and hyperammonemia in CTLN2, so that low carbohydrates diet is important. High carbohydrates and low protein diet is the common therapeutic diet for liver diseases, which, however, was reported inappropriate [65]. Rather, carbohydrate-restricted diet or in addition high protein diet were found to be effective [2,66]. Infusion of high glucose solutions [67] or hyperosmotic solutions [62,68] for brain edema, such as Glyceol consisting of 10% glycerol and 5% fructose is contraindication. Infusion of high concentration of glucose caused hypermmonemia and consciousness disturbance to citrin deficiency patients [67]. You can use lower concentration (less than 10%) of glucose, if the patients needed glucose. You may find many case reports listed in reference [62]; many case-report authors described that patients given Glyceol got irreversible deterioration and mostly died shortly after infusion of Glyceol [62].

**Figure 6:**
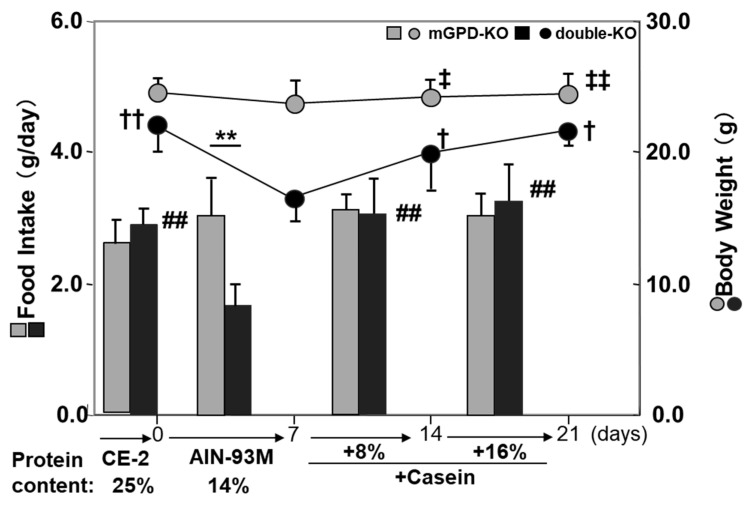
Intake of diet (high carbohydrate diet with defined composition, AIN-93M (recommended for mature rodent by American Institute for Nutrition in 1993) and body weight changes per week of mGPD-KO and double-KO mice and effect of supplementation of casein. The mice were usually given a high protein diet CE-2 (25%) from Clea Japan and changed the diet to AIN-93M (protein content is 14%), the intake is the average of a week and body weight changes in a week were monitored. Supplementation was done with reducing corn starch by the same amount. Differences in body weight for double-KO mice († *p* < 0.05, †† *p* < 0.01), and for mGPD-KO mice (‡ *p* < 0.05; ‡‡ *p* < 0.01) comparing the AIN-93M diet versus other supplementation were determined using a paired t-tests. Difference in food intake between diets within each genotype were determined using paired t-tests (# *p* < 0.01; ## *p* < 0.01) Difference in food intake between genotypes were determined using unpaired t-test (** *p* < 0.01) From [71].

On the other hand, we noticed that protein and some kinds of lipid in the diet may be effective for treatment. Nutritional assessment of a young girl with CTLN2 [69] disliked high carbohydrates diet and sweets, and she took her favorite diet which was rich in protein and lipid in spite of that with almost the same amount of carbohydrates as the high carbohydrate diet she took, suggesting an ameliorating effect of the other nutrients [70]. In the double-KO mice with component-defined diet, we showed that the high-carbohydrate diet (AIN-93, a diet for mature rodent recommended by American Institute for Nutrition in 1993) was strongly avoided by the double-KO mice, but addition of 8% protein (casein) by reducing carbohydrates by 8% caused appetite enough to recover the amount intake as usual with high protein laboratory chaw (CE2, a diet for rodent breeding supplied from CLEA Japan) [71], as shown in Figure 6. The casein was able to be replaced by single amino acid such as Ala and Glu. Furthermore, addition of 6% MCT (medium-chain triglycerides) alone caused full intake as CE2, but none of triglycerides which consisted from long-chain fatty acids such as soybean oil, fish oil, and lard were effective, as shown in Table 2. It is because MCT is metabolized in the liver but not via tightly-regulated carnitine palmitoyltransferase I pathway.

**Figure 7:**
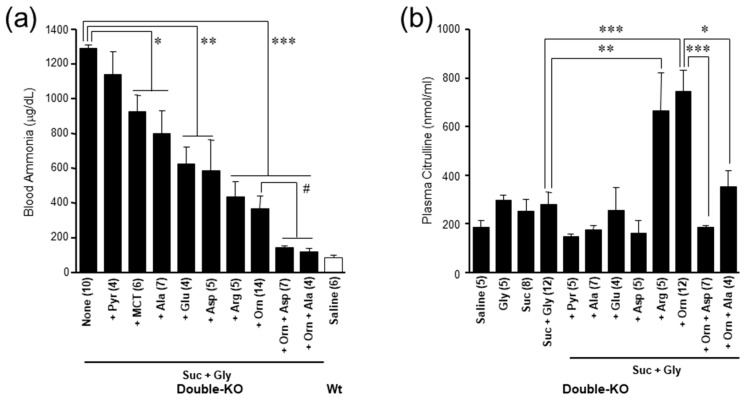
(**a**) Effects of enteral administration of 0.5 M various amino acids, 0.5 M sodium pyruvate, 5% MCT, or amino acid combination (10 mL/kg bw) on blood ammonia increased by administration of 20% sucrose +1 M Gly (20 mL/kg bw) in double-KO mice. (**b**) Effects of administration of amino acid and other substances indicated in the figure on plasma citrulline in the double-KO mice. Asterisks (* *p* < 0.05; ** *p* < 0.001; *** *p* < 0.0001) denote statistical differences between indicated groups by unpaired t-test (Figure 7(a)) Asterisks (* *p* <0.05; ** *p* < 0.001; *** *p* < 0.0001) denote statistical differences vs saline. Values are shown with mean±standard error of mean. From [72].

From the results, we searched for the most effective amino acid(s) [72]. Looking for amino acids which increase hepatic Asp, or decrease hepatic G3P level increased by administration of ethanol to citrin-KO mice [72]. Resultant candidate amino acids were glycine (Gly), Ala, ornithine (Orn), arginine (Arg), serine, Asp, and Glu. During this research, we also looked for amino acids which affect the hepatic citrulline level. Gly and serine were found to increase the hepatic citrulline level and furthermore, blood ammonia levels. According to this finding, we eliminated Gly and serine from the list. Furthermore, we were able to create a severe hyperammonemia model by administration of Gly and sucrose to the double-KO mice [72]. Using this model, we finally decided which amino acid(s) are the most effective. Figure 7a shows you the effect of various amino acids and MCT on decreasing blood ammonia increased by the addition of sucrose and Gly. The most effective were Orn+Asp or Orn+Ala; those decreased blood ammonia as low as wild type mice without Suc + Gly. Figure 7b shows plasma citrulline levels. Orn is well known to stimulate urea synthesis by increasing urea cycle capacity through increasing substrate level of OTC reaction, but increased plasma citrulline level as Arg (Figure 7b). Addition of Ala or Asp to Orn decreased the plasma citrulline level, indicating the action of these two amino acids are different from Orn and Arg. We postulated the acting point of Glu, Asp, and Ala are the same.

**Figure 8:**
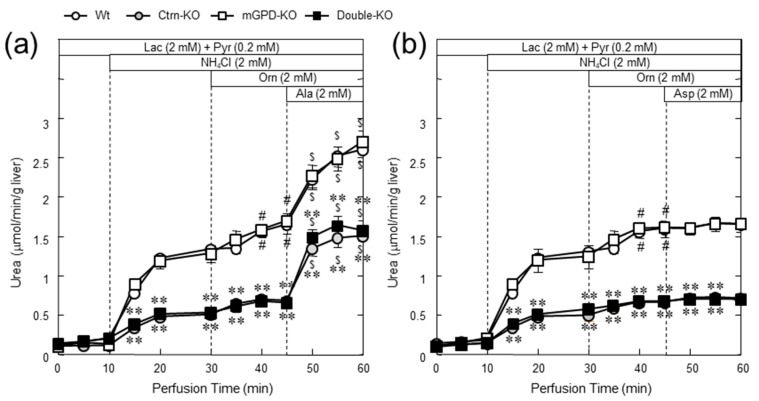
Ureagenesis from ammonium chloride in the perfused liver and effect of Ala (**a**) and Asp (**b**). In the upper part of the figures, the addition to the perfusion media is shown. Mice used are, wild type (white circle), citrin-KO (gray circle), mGPD-KO (white squire), and double-KO mice (black squire). * and ** indicate statistical differences (*p* < 0.05 and *p* < 0.01, respectively) from wild type values and # and $, *p* < 0.05 from the level at perfusion time 45 and 30 min within the same genotypes. From [72].

On the other hand, we also tested Ala and Asp on ureagenesis in the perfused liver [72], as shown in Figure 8. The results show that Ala was effective in ureagenesis in any kinds of genotype mice including the double-KO mice but Asp was not at all effective. We have also reported the similar results with citrin-KO mice showing ineffectiveness of Asp [73]. This is accordant with the report by Stoll et al. [74] that Asp together with Glu and α-ketoglutarate administered are transported into perivenous hepatocytes but not into periportal hepatocytes where the urea cycle enzymes are located. However, it is not consistent with our results showing effectiveness of Asp per os administered, as shown in Figure 7a.

## 9. Importance of Small Intestine on Metabolism of Amino Acids

**Figure 9:**
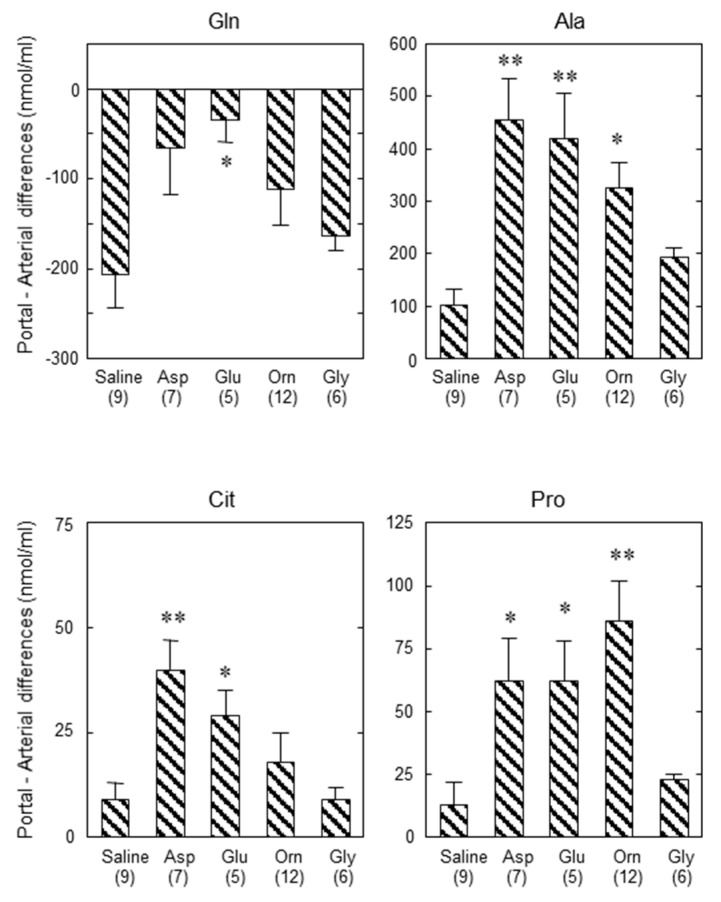
Portal vein-arterial differences in the plasma concentration of Gln, Ala, Cit, and Pro 1 h after administration of saline, Asp, Glu, Orn, or Gly. Plus values indicate output and minus values indicate uptake of the amino acid. Saline, Asp, Glu, Orn, or Gly (20 mL/kg bw; 10 mmol/kg bw) were enterally administered to mGPD-KO mice and 1 h after the administration, blood was collected simultaneously from portal vein and abdominal aorta for portal vein-arterial difference. * *p* < 0.05 and ** *p* < 0.001 vs saline. From [70].

**Figure 10:**
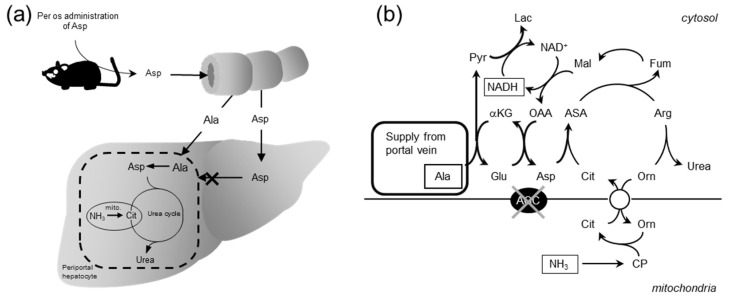
Schematic diagram of Asp metabolism after enteral administration within the small intestine (**a**) and liver (**b**) and postulated metabolic pathway of Ala in periportal hepatocytes. From [72].

**Figure 11:**
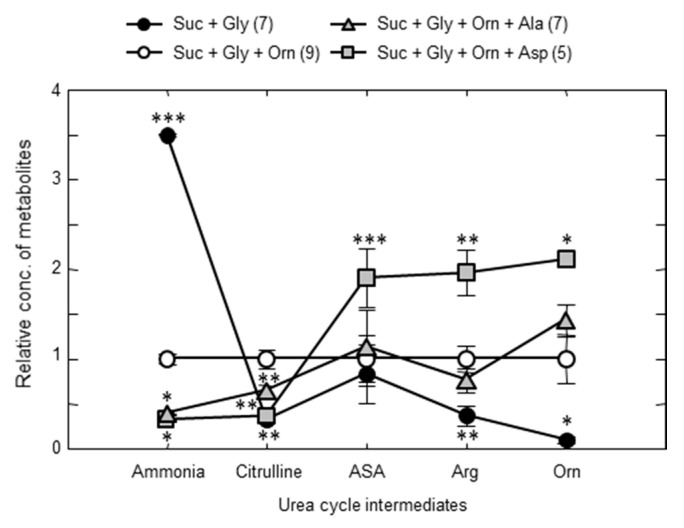
Crossover point analysis of Asp action. Each of hepatic and blood metabolite concentrations under Suc + Gly + Orn was set at 1 and the metabolite concentrations under the other conditions were calculated and plotted. The concentration of blood ammonia was used as ammonia in the figure. The line formed with Asp crossovered the basal line between citrulline and argininosuccinate, indicating that Asp activates ASS step. * *p* < 0.05; ** *p* < 0.001 and *** *p* < 0.0001 from the baseline of Suc + Gly + Orn. From [72].

To solve this discrepancy, we considered the role of the small intestine in metabolism of amino acids. Actually, Neame and Wiseman [75], Parsons and Volman-Mitchell [76], and Windmueller and Spaeth [77] have shown that enterally-administered Asp is converted into Ala in the small intestine and then transported via the portal vein to the liver. In order to examine the role of the small intestine on the function of amino acids in the liver and confirm the results shown by the former researchers, we administered various amino acids enterally and assayed the concentrations of amino acids in the portal vein and abdominal aorta and calculated the difference in the amino acids between the two blood vessels. In our experiment, enteral administration of specific amino acids caused increases in each amino acid within the arterial circulation, and the clear portal-arterial difference, demonstrating an uptake of Gln and output of Ala, citrulline, and Pro (Figure 9). Our findings clearly demonstrate that some part of the Asp, Glu, and Orn were converted to Ala, but no significant conversion of Gly to Ala in the small intestine (Figure 9). It also suggests that the positive effect of Orn on enhancing ureagenesis appears to be more than simply increasing the urea-cycle intermediate levels. The results shown here suggest also that the Ala formed and supplied to the periportal hepatocytes can then be converted back to Asp by coupling of two cytosolic aminotransferases (Figure 10a,b), and the formed pyruvate can then oxidize malate to oxaloacetate, reinitiating ureagenesis under citrin deficiency (Figure 10b). Actually, the combination of Orn and Asp, L-ornithine L-aspartate (LOLA), which contains no NaCl, was very effective in decreasing blood ammonia [72]. Acting site of Asp, analyzed by using crossover point analysis, clearly is an ASS step, as shown in Figure 11. This will be effective therapeutics for citrin deficiency. We are now examining LOLA as a clinical test.

## 10. Drugs or Supplements Used as Therapeutics for Citrin Deficiency at Present

**L-Arginine**: Arg was the first drug found effective in the blood ammonia decreasing drug by Hoshi et al. [78] and Imamura et al. [66]. It was effective at not only decreasing blood ammonia but also effective in decreasing plasma triglyceride [66].

**Sodium Pyruvate:** Sodium pyruvate was used to consume glycolytic NADH by producing lactate, and then decreasing blood ammonia probably by producing oxaloacetate and aspartate. It was found effective in controlling the pathogenic state of citrin deficiency by Moriyama et al. [73], clinically by Mutoh et al. [69] and Yazaki et al. [79].

**MCT:** MCT was originally used as an energy supply for various metabolic diseases including NICCD [80]. Hayasaka et al. [81] successfully used MCT for the treatment of CTLN2. They emphasize MCT as the energy source for the liver. But the mechanism of action is not clear yet. Saheki et al. [71] used MCT in the treatment of model mice, and Saheki et al. [82] and Moriyama et al. [83] analyzed the mechanism of action of MCT, the latter using the perfused liver system. Both agreed MCT increased synthesis of Gln. How does Gln, which is not the end product, but an intermediate metabolite of nitrogen metabolism work? Therefore, how Gln works by further metabolism is the next question.

All these clinical tests propose effectiveness of Arg, sodium pyruvate, and MCT in treatment of citrin deficiency. Further extensive clinical trials of these candidates including LOLA are needed.

## Figures and Tables

**Table 1:** Two distinct types of citrullinemia.

Type of Enzyme Abnormality We Named:	Type I	Type III	Type II
ASS activity or enzyme amount:	Abnormal kinetics	Almost null	Decrease of normal ASS
Organ specificity	All cells	All cells	Liver-specific
Type of abnormality	Qualitative		Quantitative
Disease:	CTLN1 (Classical citrullinemia)		CTLN2 (Adult-onset type II citrullinemia)
Gene:	*ASS1*		*SLC25A13* (Citrin deficiency)

**Table 2:** Effect of supplementation of protein, amino acids, and lipid on the food intake and body weight changes as shown in Figure 6. From [71].

Change from CE-2 to	Body Weight	Food Intake
AIN93M	Dec	Dec
(Effect of supplements)		
Protein (+8%)	Inc	Inc
Ala (5%)	Inc	Inc
Na-Glu (5%)	Inc	Inc
Na-Pyr (10%)	Inc	Inc
MCT (6%)	Inc	Inc
Soybean oil (6%)	no	no
Lard (6%)	no	no
Olive oil (6%)	no	no
Fish oil (6%)	no	no

Inc, increased; Dec, decreased; No indicates no change.
